# Race analysis in swimming: understanding the evolution of publications, citations and networks through a bibliometric review

**DOI:** 10.3389/fspor.2024.1413182

**Published:** 2024-06-13

**Authors:** Jorge E. Morais, Tiago M. Barbosa, Raul Arellano, António J. Silva, Tatiana Sampaio, João P. Oliveira, Daniel A. Marinho

**Affiliations:** ^1^ Department of Sports Sciences, Instituto Politécnico de Bragança, Bragança, Portugal; ^2^ Research Centre for Active Living and Wellbeing (LiveWell), Instituto Politécnico de Bragança, Bragança, Portugal; ^3^ Aquatics Lab, Department of Physical Education and Sports, Faculty of Sport Sciences, University of Granada, Granada, Spain; ^4^ Research Centre in Sports, Health and Human Development (CIDESD), Covilhã, Portugal; ^5^ Department of Sports Sciences, University of Trás-os-Montes and Alto Douro, Vila Real, Portugal; ^6^ Department of Sports Sciences, University of Beira Interior, Covilhã, Portugal

**Keywords:** swimming, trends, bibliometric data, race analysis, performance

## Abstract

The aim of this study was to conduct a scoping and bibliometric review of swimming articles related to race analysis. The Preferred Reporting Items for Systematic Reviews and Meta-Analyses (PRISMA) guidelines were used to identify relevant studies. Articles on race analysis in swimming published between 1984 and December 31, 2023 were retrieved from the Web of Science database. 366 records were screened and a total of 74 articles were retained for analysis. Until 2012, there were some time intervals with no or few publications. From 2012, there was a clear upward trend in publications and citations. This theme was led by the United States of America, Australia, and Spain. Australia and Spain maintain their status as the countries with the most publications. The analysis of author collaborations revealed two clusters with Spanish authors, and the remaining clusters are composed of Portuguese, Swiss, and Australian authors. With this bibliometric review, it has been possible to understand the evolution of the articles published on race analysis in swimming, the countries and the authors that have contributed most to this topic over the years. The prediction model shows that the number of articles and citations on this topic will continue to increase over the next 10 years (until 2034).

## 1 Introduction

Swimming is a time-based sport where improved performance is strongly dependent on the holistic interaction of several determinants from different scientific fields (e.g., anthropometrics, motor control, biomechanics, energetics/efficiency) (1). Experimental (2, 3) and numerical (4, 5) research in swimming has helped to better understand these findings and how to apply them in a training context to improve performance. However, this type of research tends to be more limited or restricted in terms of the number of participants and their level of performance (6). In fact, even today, experimental studies of swimming performance tend to recruit small numbers of swimmers, usually at the national level, and adolescent or young adult swimmers (7, 8). On the other hand, race analysis from official competitions (national or international championships or Olympic Games) allows to analyze: (i) a larger number of swimmers; (ii) elite level swimmers, and; (iii) analyze these swimmers in a real competition scenario (6, 9). These analyses are important for both swimmers and coaches to understand the behavior of the latter in real competition scenarios, so that swimmers can understand how to improve for future competitions.

To the best of our knowledge, only one study has conducted a narrative review study of race analysis in swimming to highlight the knowledge on this topic to date (10). Usually, studies on this topic focus on the performance times and kinematic variables (spatiotemporal) related to the swimmers' start, swim stroke, turns, and finish (11–13). Up to that point, it was noted that swimming research on race analysis had focused primarily on long-course, adult/elite, and 100- and 200-meter events (10).

The review studies (systematic, narrative, or scoping) perform a quantitative or qualitative data analysis by synthesizing the results of the selected studies and drawing conclusions based on the overall body of evidence (14). These “traditional” reviews focus on identifying, selecting, and synthesizing all published research on a particular topic. Conversely, bibliometric reviews are a rigorous process for evaluating large amounts of scientific information to provide meaningful interpretations of research topics and future trends (15, 16). They offer a unique perspective by systematically analyzing publication trends, key contributors, and emerging clusters in the step test literature (17). They can identify the countries, journals, institutions, and authors that are most active in each research area, as well as existing collaborations between authors, institutions, and countries. Thanks to bibliometric studies, researchers can master the literature in a short time by reading the abstracts obtained from the analysis of hundreds of articles from the past to the present (18, 19).

Bibliometric reviews have been used in several fields of research, such as linguistics (19), medicine (20), or environmental health (21). In the case of sport, studies can be found related to coaching leadership (22), sport management (23), or sport education (24). However, there are few studies on sport performance (25, 26) and only one in swimming (27). A bibliometric review of race analysis in swimming can provide insights into collaborative networks among researchers, institutions, and countries. This information is valuable for understanding the global landscape of scientific collaboration on this topic. Therefore, the aim of this study was to perform a scoping and bibliometric review of swimming articles related to race analysis. It is expected to better understand the chronological distribution of publications and citations, the countries/regions and institutions network, and the journal and authors that have contributed most to this topic.

## 2 Methods

### 2.1 Data source and search strategy

The Web of Science (WoS) is a collection of reliable global citation databases covering more than 250 fields and all regions (28). The WoS list provides extensive information on definitions, coverage notes, and the most significant impact factor score for various journals selected based on the index (28). It has been widely used in previous bibliometric studies, including sports (24, 25). Therefore, the WoS Core Collection (WoS by Clarivate Analytics) database was used in this bibliometric review.

The search spanned until December 31, 2023, and two strategies were developed to account for the recent emergence of the topic and the different nomenclature used by authors in the field. Thus, the search strategies were as follows: (i) (((TS = (“swimming”)) AND TS = (“race analysis”)) OR TS = (“swimming”)) AND TS = (“pacing”) and (ii) ((TS = (“swimming”)) AND TS = (“race”)) AND TS = (“analysis”). The decision to use two different strategies was based on the recognition that authors exploring race analysis in swimming may use different terms. This approach aimed to compile a comprehensive collection of articles on the topic, ensuring that the literature review captured the most relevant publications.

### 2.2 Inclusion and exclusion criteria

The following inclusion criteria were applied: (i) written in English; (ii) articles that directly addressed the topic of race analysis in swimming; (iii) articles that provided information on critical performance aspects, such as the start, turn(s), clean swim, and finish; (iv) articles that covered race analysis in any stroke technique and in different pool sizes; (v) articles that included both able-bodied and Paralympic swimmers; and (vi) articles that evaluated race analysis in official national and international swimming events.

Exclusion criteria were: (i) articles written in languages other than English; (ii) articles lacking information on critical performance aspects, such as the start, turn(s), clean swim, and finish; (iii) articles not evaluating race analysis in official swimming events; (iv) articles focusing on open water competitions; and (v) articles not retrieved or not retrieved from WoS during this period.

### 2.3 Screening process

The titles and abstracts of the selected publications were reviewed separately by two reviewers. The full text was collected in cases where the eligibility of an article was unclear. The same two reviewers examined the integral's articles and assessed the eligibility criteria. Each article underwent two rounds of independent evaluation by these reviewers. First, the title and abstract were evaluated, and then the full content of the article was evaluated. Eligibility disputes were resolved through discussion and, when necessary, with the assistance of a third reviewer.

**Figure 1:**
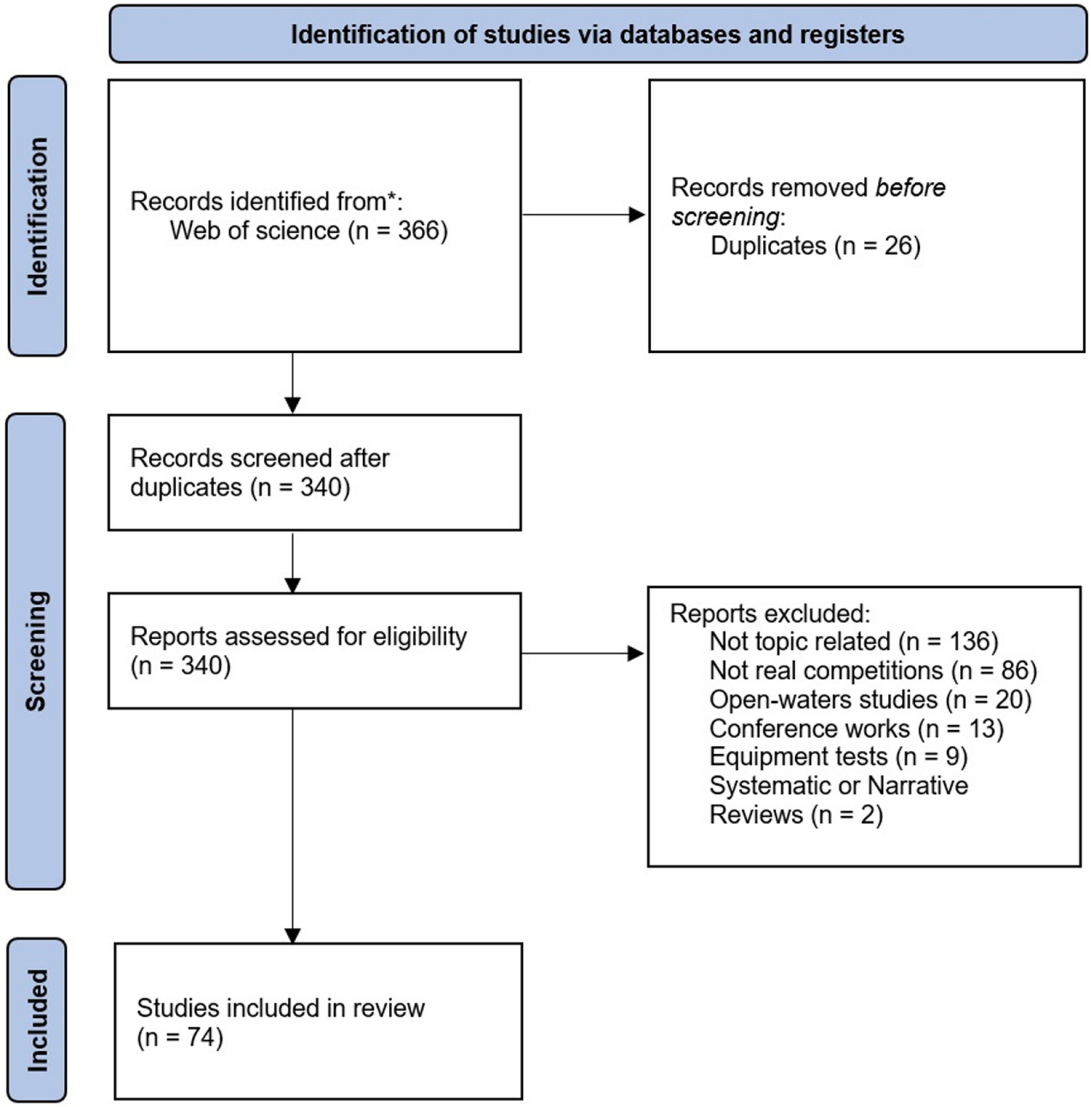
Flowchart of the review.

The WoS search yielded 366 records, of which 26 were duplicates and were therefore excluded. The remaining 340 studies were then assessed by reading the relevant sections. 265 studies that did not meet the inclusion criteria were excluded. Finally, a total of 74 articles met the defined criteria and were included in the review. Figure 1 shows the identification, screening, and inclusion of the articles from the WoS database for the review. When full-text publications were accessible, further information was retrieved for an in-depth study, including the approach and results. The 74 articles included in this review are listed in Table 1. This includes information on the swimmers and the level of competition, the swimming event/stroke, and the sexes analyzed.

**Table 1:** Summary of the 74 articles included in the review. This includes information on the swimmers and the level of competition, the swimming event/stroke, and the sexes analyzed.

Reference	Sex	Level	Competition(s)	Event(s)
Arellano et al. (6)	Male and female	Olympic	50, 100, and 200 m freestyle	1992 Barcelona Olympic Games
Arellano et al. (9)	Male and female	International	50 m backstroke, breaststroke, butterfly, and freestyle	2021 Budapest European Championships
Barroso et al. (29)	Male	Elite	200 and 400 m freestyle	–
Born et al. (30)	Male	National, International and World-class	All backstroke, all breaststroke, all butterfly, all freestyle, and all medley	–
Born et al. (31)	Male	International	All backstroke, all breaststroke, all butterfly, and all freestyle	2019 European Short Course Championships
Born et al. (32)	Female	International	All backstroke, all breaststroke, all butterfly, and all freestyle	2019 European Short-Course Championships
Born et al. (33)	Male and female	International	100, 200, and 400 m short-course individual medley in all strokes	2019 European Short-Course Championships
Born et al. (34)	Male and female	All levels	All strokes within all distances	All events
Born et al. (35)	Male and female	All levels	All strokes within all distances	All events
Breen et al. (36)	Male and female	International and world-class	4 single strokes 200 m individual events	2016 Olympic Games and the LEN European Championships 2016
Burkett and Mellifont (37)	Male	World-class and paralympic	100 m freestyle	2002 World Championships, 2004 Athens Paralympic Games, and the 2006 World Championships
Chow et al. (38)	Male and female	International	All backstroke, all breaststroke, all butterfly, and all freestyle	1982 British Commonwealth Games
Cuenca-Fernandez et al. (39)	Male and female	International	100, 200, 400, and 800 (females only) freestyle/1,500 m (males only) freestyle	2019 European Short-Course Championship
Cuenca-Fernandez et al. (40)	Male	International	100 and 200 m	2019 European Short-Course Championships
Daly et al. (41)	Male	Paralympic	100 m freestyle	1996 Atlanta Paralympic Games
Demarie et al. (42)	Male	International, world-class, and olympic	1,500 freestyle	Long course finals of the Olympic Games 2021 (Tokyo 2020), FINA World Championships 2022 (Budapest), short course finals FINA Swimming World Cup 2021 (Berlin), and FINA Swimming World Cup 2021 (Budapest)
Escobar et al. (43)	Male and female	Top-level	200 m freestyle	FINA World Championships (Kazan, Russia 2015) and the French National Championships (Montpellier 2016).
Fischer et al. (44)	Male and female	International and olympic	4 × 100 m freestyle relays	Three Olympic Games (2008, 2012, 2016) and five World Championships (2007, 2009, 2011, 2013, 2015)
Fortin-Guichard et al. (45)	Male and female	International	100 m freestyle	–
Fritzdorf et al. (46)	Male	World-class	100 m breaststroke	World Championship semi-final and final 2005, Commonwealth Games semi-final and final 2006, European Championship semi-final and final 2006 and World Championship semi-final 2007
Fulton et al. (47)	Male and female	Paralympic	100 m freestyle	Australian, British, German and American National Championships, the 2004 Paralympic Games, and 2006 World Championships.
Garcia-Hermoso et al. (48)	Male and female	International, world-class, and olympic	50, 100 and 200 m backstroke	Olympic Games (2008, 2012), World Championships (2007, 2009, 2011, 2013 and 2015), European Championships (2006, 2008, 2010, 2012 and 2014), Commonwealth Games (2006, 2010 and 2014), Pan Pacific Games (2006, 2010 and 2014), U.S. Olympic Team Trials (2008, 2012), Australian Olympic Team Trial (2012),
Gatta et al. (49)	Male	Elite	400 m freestyle	2009 Italian Swimming Championship
Gonjo et al. (50)	Male and female	International	200 m IM, backstroke, breaststroke, butterfly, and freestyle	2021 European Long-Course Swimming Championships
Hogarth et al. (51)	Male	Paralympic	50, 100 and 400 freestyle 100 m backstroke, breaststroke, and butterfly	–
Lara and Del coso (52)	Male and female	World-class	1,500 m freestyle	World Championships (2003, 2005, 2007, 2009, 2011, 2013, 2015, 2017, 2019)
Lipinska et al. (53)	Male	Regional, National, International, World-class, and Olympic	1,500 m freestyle	Olympic Games, World Championships, European Championships or Pan-Pacific Championships, International Meets, and National Championships
Lipinska et al. (54)	Female	Regional, National, International, World-class, and Olympic	800 freestyle	Olympic Games, World Championships, European Championships or Pan-Pacific Championships, International Meets, and National Championships
Lopez-Belmonte et al. (55)	Male and female	International	400, 800 and 1,500 m freestyle	2021 European Championship
Malone et al. (56)	Male and female	Paralympic	50 and 100 m freestyle	1996 Paralympic Games
Marinho et al. (57)	Male and female	International	100 and 200 m backstroke, breaststroke, butterfly, and freestyle	2018 long course meter LEN European Aquatics Championships
Mauger et al. (58)	Male and female	National, International, World-class, and Olympic	400 m freestyle	British and Australian Championships, International Invitational Meets, European Championships, World Championships, and Commonwealth and Olympic Games in the period 2003–2010
McCabe et al. (59)	Male and female	World-class and Olympic	50, 100 and 200 m breaststroke	World Championships 2015, 2017, 2019 and Olympics 2016
McGibbon et al. (60)	Male	Elite	1,500 m freestyle	–
McGibbon et al. (61)	Male and female	International, World-class, and Olympic	4 × 200 m freestyle	–
Miller et al. (62)	Male and female	International	100, 200 and 400 backstroke, breaststroke, butterfly, and freestyle 200 and 400 IM	1982 British Commonwealth Games
Morais et al. (63)	Male	International	50 m freestyle	2019 Long-Course Meter LEN European Junior Championships, 2021 LEN European Championships
Morais et al. (64)	Male	International	1,500 m freestyle	2016 and 2018 LEN European Aquatic Championships
Morais et al. (65)	Male	International	100 m freestyle	2019 LEN European Junior Championships
Morais et al. (66)	Male	International	50 m backstroke, breaststroke, butterfly, and freestyle	2021 LEN European Championships
Morais et al. (67)	Male	International	50 m freestyle	2019 Long-Course LEN European Junior Championships
Morais et al. (11)	Male and female	International	100 m backstroke, breaststroke, butterfly, and freestyle	2016 LEN European Championships
Moser et al. (68)	Male and female	World-class	100 and 200 m backstroke, breaststroke, butterfly, and freestyle	XV FINA World Masters Championships 2014, XVI FINA World Masters Championships 2015, XVII FINA World Masters Championships 2017, and XVIII FINA World Masters Championships 2019
Mytton et al. (69)	Male and female	International	400 m freestyle	Ligue Européenne de Natation European Championships 2008 and 2010 and the Federation Internationale de Natation World Championships in 2007, 2009, and 2011
Mytton et al. (70)	Male and female	International, world-class	400 m freestyle	LEN European Championships in 2006, 2010, and 2012; World Championships in 2007 and 2011; The Commonwealth Games in 2006
Neuloh et al. (71)	Male and female	World-class and Olympic	800 and 1,500 m freestyle	World Championships and Olympic Games between 1998 and 2016
Nicol et al. (72)	Male and female	National and International	100 and 200 m breaststroke	Australian domestic and international events
Oliveira et al. (73)	Female	International	50 m backstroke, breaststroke, butterfly, and freestyle	2021 LEN European Championships
Olstad et al. (2)	Male and female	National and International	50, 100 and 200 m breaststroke	–
Pai et al. (74)	Male and female	International	100 and 200 m backstroke, breaststroke, butterfly, and freestyle	1982 British Commonwealth Games
Perez-Tejero et al. (75)	Male and female	Paralympic	100 backstroke, breaststroke, butterfly, and freestyle	2012 London Paralympic Games
Polach et al. (76)	Male	World-class	1,500 freestyle	2019 World Championships
Qiu et al. (77)	Male and female	International	relay 4 × 100 m (freestyle, medley, and mixed freestyle) and individual 100 m (butterfly, breaststroke, and freestyle)	2017 European Junior Swimming Championships
Robertson et al. (78)	Male and female	International, World-class, and Olympic	100 and 200 m freestyle, backstroke, breaststroke, and butterfly, 200 and 400 m individual medley and 400 m freestyle	2 Olympic Games, 3 World Championships, two Commonwealth Games, 2 European Championships
Saavedra et al. (79)	Male and female	International, World-class, and Olympic	200 and 400 m Individual Medley	3 Olympic Games (2000, 2004, 2008), 5 World Championships (2003, 2005, 2007, 2009, 2011), 6 European Championships (2000, 2002, 2004, 2006, 2008, 2010), 3 Commonwealth Games (2002, 2006, 2010), 3 Pan Pacific Games (2002, 2006, 2010), 3 U.S. Olympic Team Trials (2000, 2004, 2008), and 3 Australian Olympic Trials (2000, 2004, 2008).
Sanchez et al. (80)	Male and female	National	50 and 100 m breaststroke	2019 Short-Course National Championship held in Gijón (Spain)
Santos et al. (81)	Male and female	World-class	50, 100 and 200 m freestyle, backstroke, breaststroke, and butterfly	2022 FINA World Championships
Schipman et al. (82)	Male and female	Paralympic	50, 100, 200, and 400 m freestyle; the 50 and 100 m butterfly; the 50 and 100 m backstroke; the 50 and 100 m breaststroke; and the 200 m	All International Paralympic Committee competitions and FINA competitions
Simbana-Escobar et al. (83)	Male and female	World-class	50 m and 100 m freestyle	FINA World Championships (Kazan, Russia 2015) and the French National Championships
Skorski et al. (84)	Male	National and International	200 m freestyle, butterfly, backstroke, and breaststroke, and 400 m freestyle	22 national and international events
Skucas et al. (85)	Male and female	World-class disabled	50 m backstroke	2010 and 2013 World Disabled Swimming Championships. 2010 and 2013 World Disabled Swimming Championships
Taylor et al. (86)	Male and female	World-class, Olympic, and Paralympic	400 m freestyle	World Championships, European Championships and Olympic/Paralympic Games between (2006 and 2012)
Thompson et al. (87)	Male and female	National, International and World-class	100 and 200 m breaststroke	National and International Championships
Tourny-Chollet et al. (88)	Male and female	National	200 m butterfly	1998 and 1999 French National Swimming Championships
Veiga et al. (89)	Male and female	National and Regional	100 and 200 m backstroke, breaststroke, butterfly, and freestyle	Open Comunidad de Madrid
Veiga et al. (90)	Male	National and Regional	200 m backstroke	Spanish Swimming Championships
Veiga et al. (91)	Male and female	International	100 m backstroke, breaststroke, butterfly, and freestyle	Third Open Comunidad de Madrid
Veiga et al. (92)	Male	International	200 m backstroke, breaststroke, butterfly, and freestyle	2008 Open Comunidad de Madrid
Veiga and Roig (13)	Male and female	World-class	200 m backstroke, breaststroke, butterfly, and freestyle	FINA 2013World Championships
Veiga and Roig (93)	Male and female	World-class	100 m backstroke, breaststroke, butterfly, and freestyle	FINA 2013World Championships
Veiga et al. (94)	Male and female	World-class	100 and 200 m backstroke, breaststroke, butterfly, and freestyle	FINA 2013World Championships
Wadrzyk et al. (95)	Male and female	Regional	50 and 100 m freestyle	Local Competition
Wolfrum et al. (96)	Male and female	World-class	200 and 400 m medley	FINA World Swimming Championships (2000–2011)
Wu et al. (97)	Male and female	International, World-class, and Olympic	4 × 200 m freestyle	Olympic Games (2012, 2016), Pan Pacific Championships (2010, 2014), World Championships (2011, 2013, 2015, 2017), Commonwealth Games (2014, 2018) and European Championships (2010, 2012, 2014, 2016)

FINA, Fédération Internationale de Natation Amateur; LEN, Ligue Européenne de Natation.

### 2.4 Analytical methods and tools

Dedicated bibliometric software was used to extract and analyze the bibliometric data. Van Eck and Waltman developed the Java-based measurement software VOSviewer (https://www.vosviewer.com), which focuses on the construction and visualization of bibliometric networks (17). These networks include journals, researchers, or individual publications, and can be constructed based on citation, bibliographic coupling, co-citation, or co-authorship relationships. Therefore, different colors were used to visually represent unique clusters in the cooperative network visualization and the co-sponsorship network visualization, while lines connecting nodes indicate collaborative links. In particular, colors in the average publication year graph indicate different years, which facilitates temporal analysis. In addition, the color spectrum, particularly the red hue in density graphs, also reflects the different densities, with redder hues indicating denser locations.

The first category is the evaluation of individuals (primarily authors, institutions, journals, and countries) using bibliographic data. The second category, scientific mapping, is a spatial visual representation of bibliometric networks that explores the relationships between disciplines, fields, specialties, individual articles, and authors. Thus, a thorough review of the race analysis literature and its evolution was accomplished by analyzing the previous categories in the VOSviewer software.

A manuscript's citations are strongly related to the number of years since its publication (20). In general, a paper published earlier will have more time to accumulate citations than one published more recently. As a result, raw citations are not a reliable metric for assessing publication impact. Therefore, the citation analysis in this study was normalized to account for differences in publication years, allowing for a more accurate assessment of the impact of scholarly articles. Normalization is essential in bibliometric analyses to reduce bias caused by publication date differences in citation practices (98). Therefore, the number of citations achieved by each article was divided by the age of the publication in years, which was calculated by subtracting the current year (year 2023) from the year of publication. This time-based normalization method allowed for the calculation of average citations per year since publication, resulting in a standardized metric for comparing the impact of articles across publication years. The online platform available at https://app.datawrapper.de was used to generate the world map. The estimation of the number of articles likely to be published in the next 10 years (until 2033), based on past publication trends, was calculated using Excel's exponential smoothing (Microsoft, Microsoft 365, Washington, USA). This allows time-series data to be analyzed and predictions or forecasts to be made based on historical trends.

## 3 Results

### 3.1 Progression of publications by year

**Figure 2:**
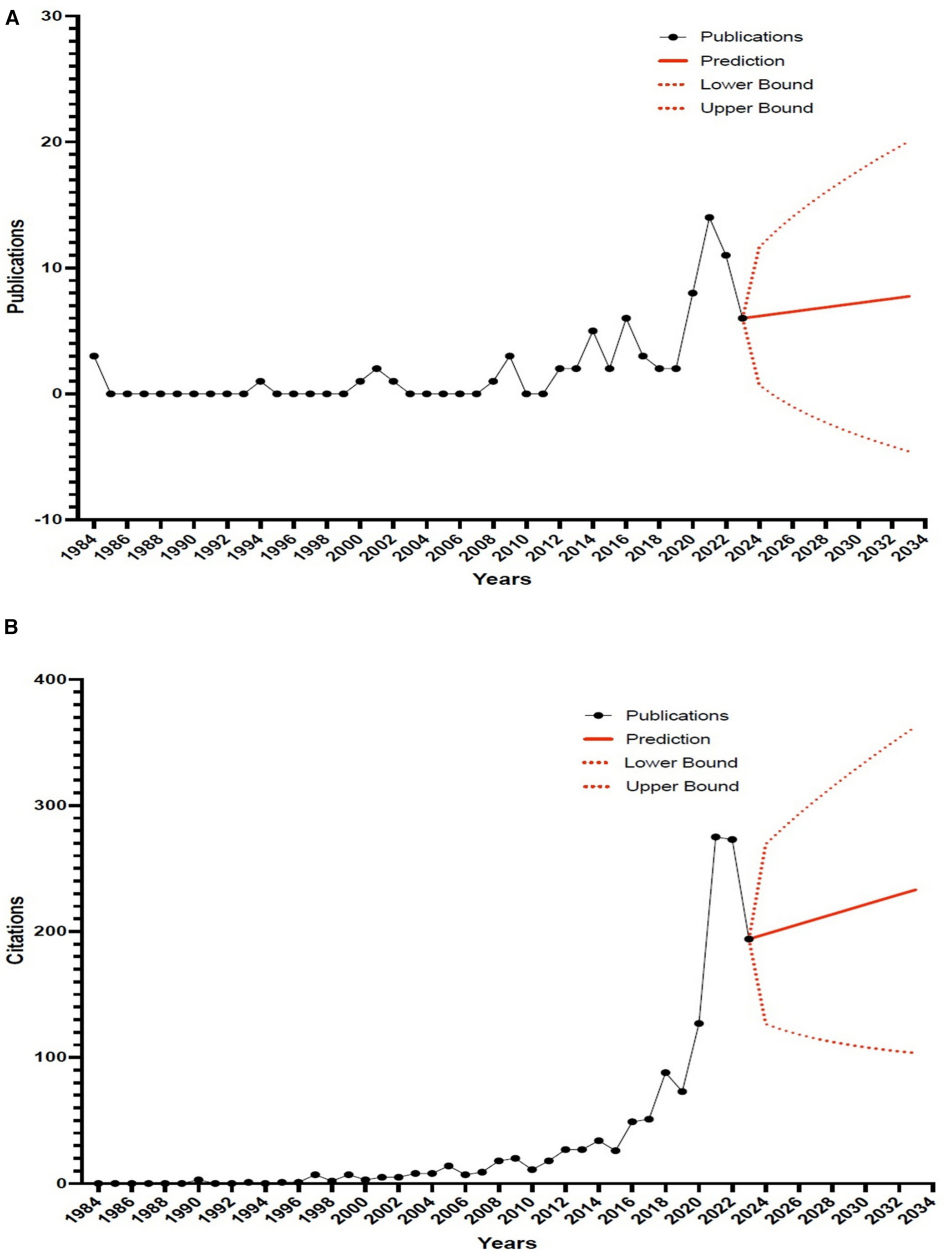
(**A**)—Publication Output on race analysis in swimming by year and estimated number of articles for the next 10 years. The red solid line represents the estimate and the red dashed lines represent the 95% confidence intervals. (**B**)—Citation outputs related to race analysis in swimming research by year and the estimated number of citations for the next 10 years. The red solid line represents the estimate and the red dashed lines the 95% confidence intervals.

Between 1984 (the year of the first publication with race analysis) and 2023, the WoS database contained a total of 74 articles related to race analysis in swimming. Figure 2A shows the publication output regarding race analysis in swimming research during the period from 1984 to 2023. Figure 2B shows the citation output regarding race analysis in swimming research during the period from 1984 to 2023. The yearly publication trends showed intermittent gaps in certain time intervals with no publications, especially in the years 1985–1993, 1995–1999, 2003–2007, and 2010–2011. Overall, there was a clear upward trend in publications on the topic until 2021. After 2021, however, there was a marked decrease in annual publications, with 14 records in 2021, 11 in 2022, and 6 in 2023. Notably, the largest number of articles was published in 2021, with 14 articles on the topic of race analysis in swimming. 2021 is also the year with the largest number of publications and citations (14 publications and 271 citations).

Publications related to race analysis in swimming showed a segmented evolution into three distinct phases, according to the number of publications: the first phase (1984–2012), the second phase (2012–2021), and the third phase (2021–2023). During this first phase, from 1984 to 2012, the field experienced intermittent publications, with sporadic activity in some years and no publications at all in others. The second phase, from 2012 to 2021, saw a notable increase in publications. After sporadic publications until 2012, the field experienced a turning point in 2014, when the number of publications exceeded six for the first time. The third phase, from 2021 to 2023, shows a reversal of the trend. While 2021 marked the peak with 14 publications, the number of publications subsequently decreased to 11 in 2022 and 6 in 2023. The exponential smoothing estimation model showed an average of 7.0 ± 0.5 articles (95% confidence intervals: −2.3–20.1) that may be published per year between 2024 and 2033. For citations, the estimation model indicated an average of 215.5 ± 11.2 citations (95% confidence intervals: 112.7–318.4) per year between the same time periods.

### 3.2 Web of science (WoS) categorization

**Figure 3:**
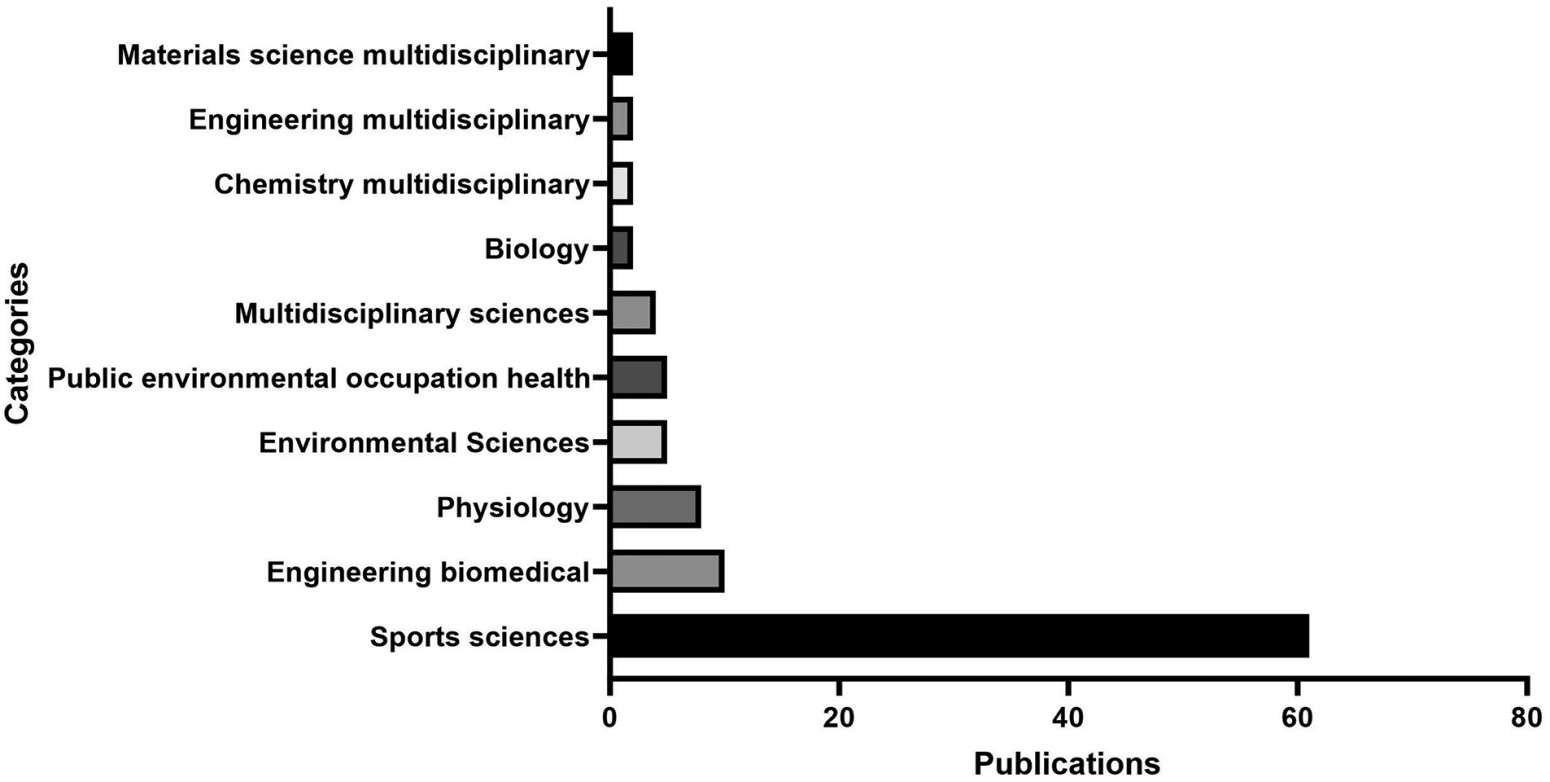
Top 10 Web of science categories for race analysis research.

By analyzing the categories within the WoS, it was possible to categorize the research field and identify possible interdisciplinary connections. Figure 3 presents the analysis of the WoS categories. The top-ranking fields are Sports Sciences (*n* = 61 publications), Biomedical Engineering (*n* = 10 publications), Physiology (*n* = 8 publications), Environmental Sciences (*n* = 5 publications), Public Environmental Occupational Health (*n* = 5 publications), Multidisciplinary Sciences (*n* = 4 publications), Biology (*n* = 2 publications), Multidisciplinary Chemistry (*n* = 2 publications), Multidisciplinary Engineering (*n* = 2 publications), and Multidisciplinary Materials Science (*n* = 2 publications). Sports Sciences emerges as the top category with 61 (81.3%) publications, underscoring its paramount importance in race analysis in the swimming research landscape.

### 3.3 Analysis of countries/regions and institutions

A total of 31 countries and regions have contributed to the analysis of race in swimming research, according to the country of the correspondent. In particular, the historical perspective highlights the development of global research. In the first decade after the publication of the first article, race analysis in swimming was led by only three countries (United States of America—USA, Australia, and Spain). Over the next two decades, however, researchers from seven countries/regions (Canada, Belgium, England, Estonia, France, Scotland, and Wales), along with the aforementioned USA, Australia, and Spain, meaningfully expanded their involvement.

**Figure 4:**
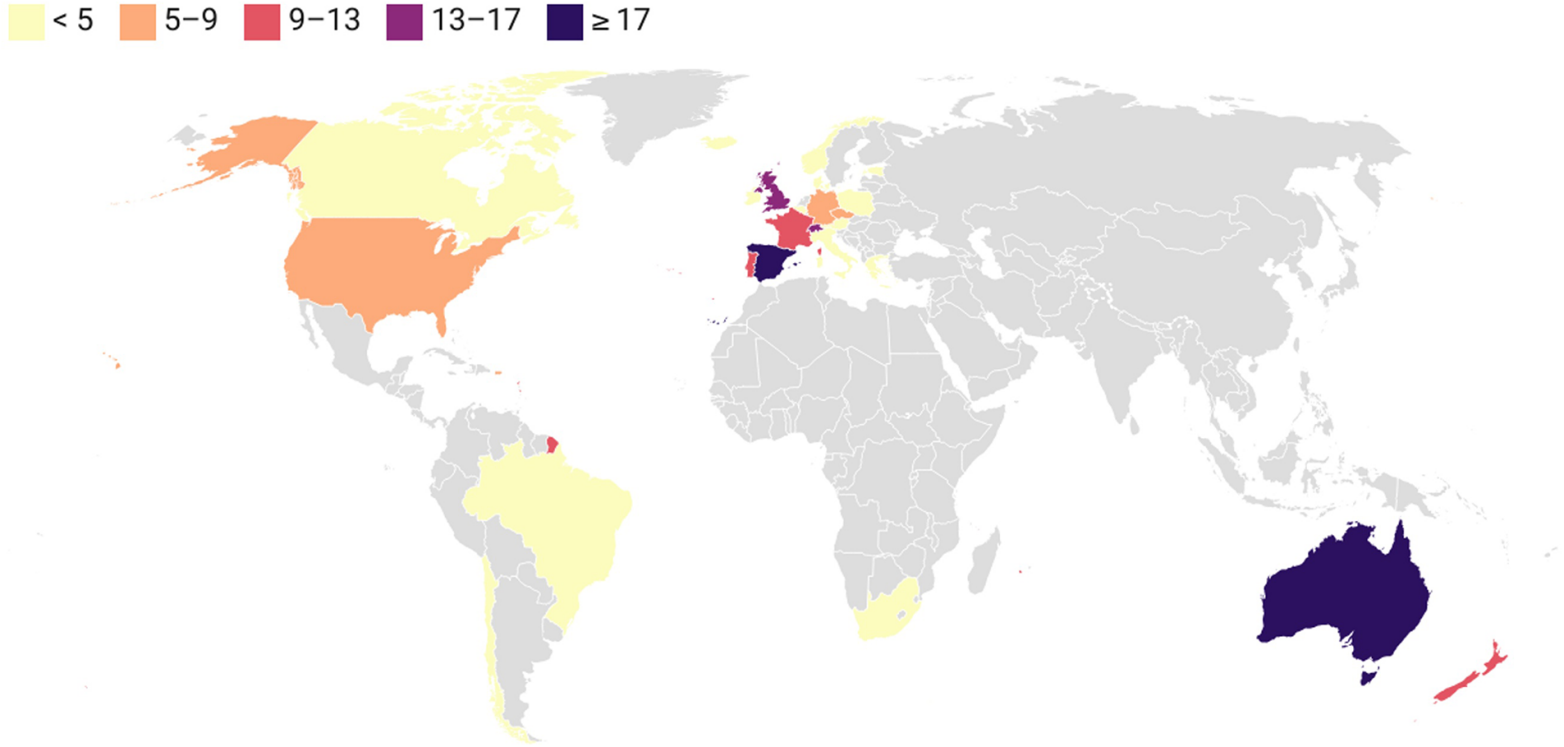
World map of the number of articles in swimming research.

The distribution of the number of articles by country/region is shown in Figure 4. The top ten countries and regions were Spain (*n* = 20 publications) with 27% of the total publications, followed by Australia (*n* = 16 publications), Switzerland (*n* = 13 publications), England (*n* = 12 publications), Portugal (*n* = 11 publications), France (*n* = 9 publications), New Zealand (*n* = 8 publications), USA (*n* = 8 publications), Czech Republic (*n* = 7 publications), and Germany (*n* = 6 publications).

Co-authorship cluster analysis, which determines the relatedness of articles based on the number of co-authored documents, was performed on 10 countries that produced at least 5 articles from the 31 countries/regions that published articles on race analysis in swimming and had international collaboration among their authors.

**Figure 5:**
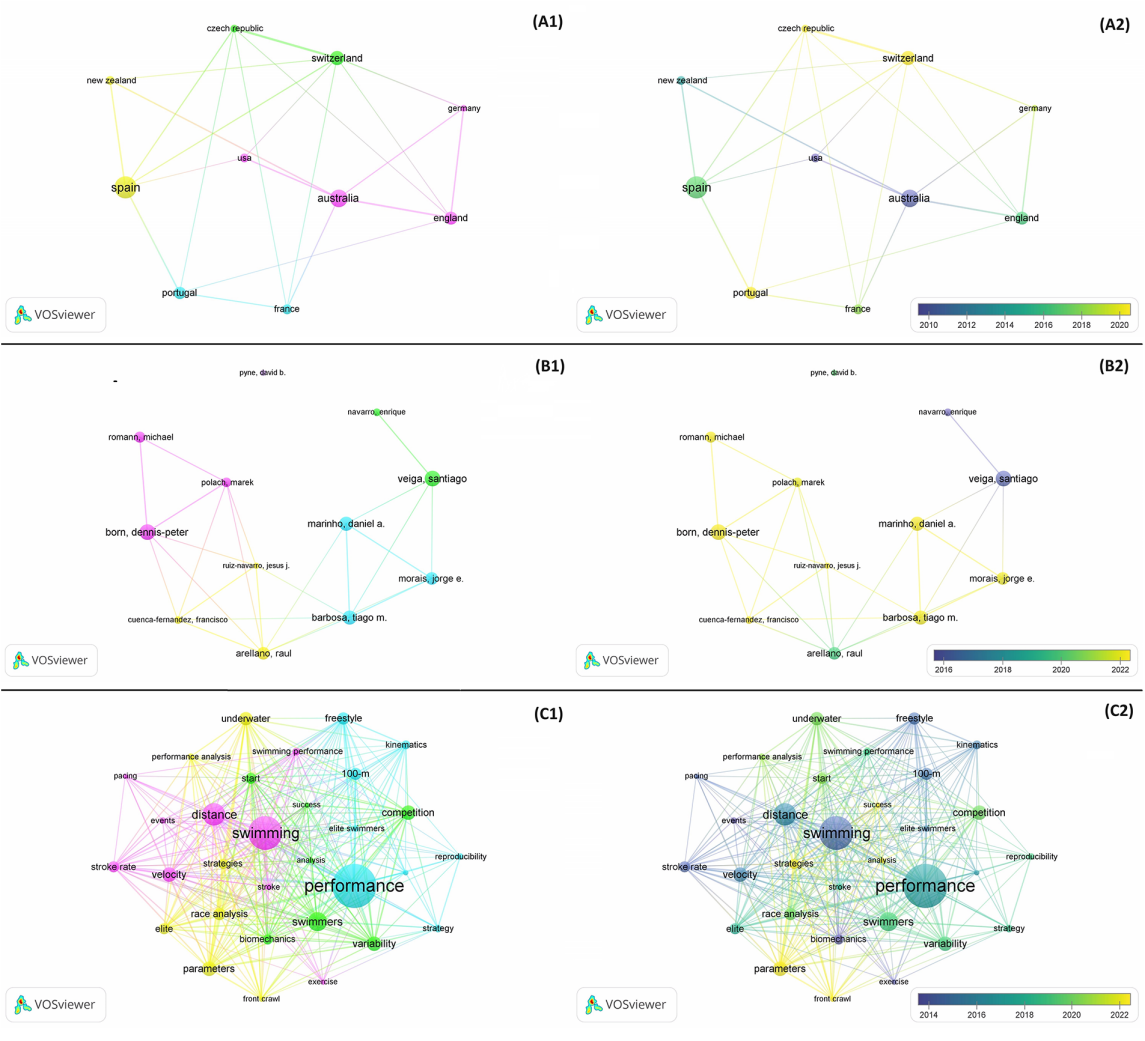
(**A1**)—The cooperation network visualization map of institutions based on VOSviewer. (**A2**)—The cooperation network visualization map of institutions based on VOSviewer with the timeline. (**B1**)—The cooperation network visualization map of authors based on VOSviewer. (**B2**)—The cooperation network visualization map of authors based on VOSviewer with timeline. (**C1**)—Keywords clustering map based on VOSviewer. (**C2**)—Keywords clustering map with the timeline based on VOSviewer.

Figure 5A shows the visualization map of the international collaboration network generated by VOSviewer (panel A1) and the visualization map of the timeline network generated by VOSviewer (panel A2). According to the results of the clustering analysis, four different clusters were formed: Cluster 1: Australia, England, Germany, and USA; Cluster 2: France and Portugal; Cluster 3: Czech Republic and Portugal; and Cluster 4: New Zealand and Spain. In addition, the total link strength scores were calculated, indicating the strength of cooperation among 31 countries. The top 10 countries/regions with the highest total link strength scores were: Australia = 14, Switzerland = 14, Spain = 11, Czech Republic = 11, England = 10, Portugal = 7, Germany = 7, New Zealand = 7, France = 6, and USA = 5.

### 3.4 Analysis of journals

A total of 26 journals were involved in publishing race analysis in swimming. The top ten journals are: *Journal of Sports Sciences* (*n* = 13 publications), *Sports Biomechanics* (*n* = 7 publications), *International Journal of Sports Physiology and Performance* (*n* = 7 publications), *International Journal of Performance Analysis in Sport* (*n* = 6 publications), *Journal of Sports Science and Medicine* (*n* = 6 publications), *International Journal of Environmental Research and Public Health* (*n* = 5 publications), *European Journal of Sport Science* (*n* = 3 publications), *Frontiers in Sports and Active Living* (*n* = 3 publications), *Applied Sciences* (*n* = 2 publications), *BMC Research Notes* (*n* = 2 publications).

### 3.5 Analysis of authors

Since 1984, 195 researchers have contributed to the advancement of research in this specific area. The use of visualization maps can provide valuable insights into potential collaborators, helping researchers to establish productive partnerships. Using a threshold of 4 documents per author, Figure 5B1 allows the visualization of five distinct clusters. As shown in the figure, the research landscape in this domain is mainly a dense network. The presence of an 11-member team participating in the collaboration is noteworthy, as highlighted in Figure 5B1. Visual inspection reveals that eight of them will be actively collaborating until December 31, 2023 (Figure 5B2. The top 10 most active authors, based on the number of documents, are Born DP (*n* = 10), Veiga S (*n* = 10), Arellano R (*n* = 9), Barbosa TM (*n* = 9), Marinho DA (*n* = 9), Morais JE (*n* = 8), Roman M (*n* = 7), Polach M (*n* = 6), Cuenca-Fernández F (*n* = 5), and Navarro E (*n* = 5).

### 3.6 Analysis of key words

Co-occurrence clustering of keywords can help identify emerging trends and patterns in the development of a topic, as well as hot areas in the field of study. It can reveal the research frontier of the field and the internal organization of an academic field. Figure 5C shows the co-occurrence keyword analysis. By using a threshold of at least five occurrences per keyword, it is possible to visualize distinct clusters. The larger the dot, the more occurrences and the more representative of the hotspots in the field. The nodes are connected to represent the strength of the association, and the more lines represent the more occurrences of two keywords in the same article. The different colors represent different clusters, i.e., research topics, and the time of appearance is represented from blue to yellow.

Therefore, the clusters emerged with the following keywords: cluster 1 (distance, events, exercise, pacing, stroke, stroke rate, swimming, swimming performance, velocity); cluster 2 (100 m, elite swimmers, freestyle, kinematics, performance, reproducibility, strategy, variables); cluster 3 (analysis, biomechanics, competition, start, success, swimmers, variability); and cluster 4 (elite, front crawl, parameters, performance analysis, race analysis, strategies and underwater). Table 2 shows the link strength of each keyword and the number of occurrences.

**Table 2:** Link strength and number of occurrences of keywords in swimming research.

Keyword	Occurrences	Total link strength
Performance	39	187
Swimming	30	127
Distance	20	115
Swimmers	17	92
Parameters	13	89
Race Analysis	11	68
Variability	13	68
Underwater	12	67
Velocity	13	65
Start	10	64
Freestyle	11	59
Stroke Rate	10	58
100-m	11	55
Elite	10	54
Competition	13	53
Strategies	9	52
Biomechanics	9	42
Front Crawl	5	42
Kinematics	8	39
Stroke	6	37
Performance Analysis	6	34
Drag	6	33
Swimming Performance	7	33
Analysis	5	27
Strategy	7	27
Variables	5	26
Success	5	23
Elite Swimmers	6	21
Events	6	20
Exercise	5	17
Pacing	5	16

### 3.7 Analysis of references

Since 1984, publications in this area have been cited 1,631 times, with an average of 15.23 ± 13.09 citations per year. The most cited article is (6) with a total of 139 citations. The top five normalized cited references are detailed in Table 3, which shows the average citations per year for each article. Notably, the article with the highest normalized citation value is by the authors Morais JE, Barbosa TM, Forte P, Bragada JA, Castro FADS, and Marinho DA with the article entitled “Stability analysis and prediction of pacing in elite 1,500 m freestyle male swimmers” (64).

**Table 3:** The five most cited references in swimming research, with the average number of citations per year for each article (citation normalization).

Rank	Title	First author (year)	Citation normalization
1.	Stability analysis and prediction of pacing in elite 1,500 m freestyle male swimmers	Morais et al. (2023) (14)	21
2.	Start and turn performances of elite sprinters at the 2016 European Championships in swimming	Morais et al. (2019) (11)	17
3.	Profiling of elite male junior 50 m freestyle sprinters: Understanding the speed-time relationship	Morais et al. (2022) (67)	15
4.	Comparison of the Start, Turn and Finish Performance of Elite Swimmers in 100 m and 200 m Races	Marinho et al. (2020) (57)	12.7
5.	Analysis and influence of the underwater phase of breaststroke on short-course 50 and 100 m performance	Sanchez et al. (2021) (80)	8.5

## 4 Discussion

The aim of this study was to perform a scoping and bibliometric review of swimming articles related to race analysis. During the period analyzed, there were some intervals without any publication. However, from 2012 onwards there was a clear sustained publication process in this topic, which reached its peak in 2021. Several decades ago, advances in technology and its access made it possible to record sports events, including swimming. Since then, coaches and athletes have begun to recognize the importance of reviewing such videos to analyze technique, identify strengths and weaknesses, and improve performance (99). This increases the role of the swimming analyst within the overall team (100). In particular, since 2013, swim race analysts/researchers have begun to use special filming setups to perform race analysis with additional kinematic data rather than just using race and split times (43, 91, 92). There have also been research groups that have performed race analyses based on video footage obtained and approved by official European organizers (11, 67). These analyses provided deeper insights into the swimmers' behavior in real competition contexts regarding all phases of the swim race, thus adding to the body of knowledge on this topic (57, 65). As a result, the number of citations was based on the number of articles published. With more information available, other researchers could better explain and interpret their findings. In addition, exponential smoothing, which is used to understand future trends in both publications and citations, indicates a continued increase over the next 10 years. Studies of race analysis in swimming have argued the importance of this type of article in understanding the pacing behavior of swimmers in various events and in defining race strategies (10, 101).

Extending the categorization within the WoS database, the implications of these classifications in the context of race analysis in swimming research were analyzed. The categorization provides insight into the research and highlights the interdisciplinary nature of the field. Sports science emerged as the dominant category, accounting for 81.3% of the publications examined. Notable examples include articles such as (55) (63), and (73), which represent the pivotal role of sports science in the study of various dimensions of swimming performance, ranging from biomechanical analysis to training methodologies and performance evaluation. Furthermore, the presence of interdisciplinary connections is evident, as evidenced by articles such as (43) and (72) in biomedical engineering (30), in physiology, and (39) and (50) in environmental sciences. These interdisciplinary connections represent collaborative efforts and diverse perspectives that contribute to the advancement of knowledge in race analysis within swimming.

The present results show that 31 countries have contributed to the topic of race analysis in swimming by the end of 2023. While initially only the USA (62), Australia (74), and Spain (6) published articles on race analysis, other developed countries also contributed to the development of this topic. Nevertheless, it must be emphasized that Spain is still the country with the highest number of publications. Looking at the topic of swimming in a broader sense, i.e., including all subtopics related to swimming (e.g., performance, biomechanics, training, etc.), it was found that the same countries are the most active overall (USA, UK, Australia, Brazil, France, Portugal, and Spain) (27). Therefore, it can be said that these countries are the ones that contributed strongly to the knowledge of swimming. Regarding the co-authorship cluster analysis, the highest total link strength score was observed for Australia and the lowest for the USA. However, when referring to the last years, Spain emerges as the most cooperative country in publishing articles on swimming race analysis. In fact, from the analysis of the cooperation of the authors (which revealed five different clusters of authors), two are composed of Spanish authors. The others are composed of Portuguese, Swiss, and Australian authors. Moreover, considering the timeline, most of the Spanish and Portuguese clusters are still actively collaborating in recent years. As it happened in a broader context of swimming research (27), it seems that Portuguese researchers are also the most active researchers in this specific topic.

The results of the co-occurrence clustering of the keywords used in the reviewed articles show that “performance”, “swimming”, and “distance” are the most used keywords. However, it should be highlighted that the keywords “parameters”, “strategies”, “front-crawl”, “analysis”, and “success” are the most used in recent years. This keyword aspect is very important for this type of analysis. For example, based on the articles retained for analysis, it was found that some articles of the present research group were not selected for analysis (102, 103). This occurred because the selection of words in the title and keywords of the articles were not related to the chosen search strategy on this topic (please refer to the subsection “Data source and search strategy”). This highlights the importance of including specific words in the title and keywords of the articles so that readers who want to search for articles on a given topic can find them. Indeed, this co-occurrence clustering of keywords can provide insights into emerging trends and patterns in the evolution of a given topic (104). As for the articles with more references during the period analyzed, the article by Arellano and co-workers (6) is the most cited with 139 raw citations. However, when the normalization of citations is reported, the research group of Morais and co-workers appears with the three most referenced articles until the end of 2023 (11, 64, 67). As mentioned above, this normalization process is essential to reduce the bias caused by publication date variances. This allows a deeper insight into the articles that have a stronger impact in the literature. However, it should be mentioned that although this type of standardization is correct and used, it could positively bias the most recent publications compared to the previous ones. In fact, this topic was not frequently published until 2012. In the results section, the top ten journals that have published articles related to race analysis in swimming are listed. Therefore, it can be suggested that authors who want to publish articles on this topic should target such journals. However, one can argue about the effect of open access journals that may have influenced the distribution of publications in other journals. For example, studies published in the International Journal of Environmental Research and Public Health, which is not specific to Sports Sciences area, but has a good impact factor and accepts studies on this topic. In addition, and in relation to swimming, it was found that the journals in which these articles are published also tend to be the most influential, according to the average number of citations per published article (27). Race analysis in swimming is still a topic to be developed compared to other topics in swimming. It is therefore logical that the journals that publish such articles are also the ones that are cited the most.

Based on the findings of both the categorization analysis and the keyword co-occurrence analysis, the present study reveals potential research hotspots within the field of race analysis in swimming. The categorization analysis, as represented in the WoS database, highlights sports sciences as the dominant category, indicating a focus on performance metrics and training methods. This is consistent with the emergent themes identified in the keyword co-occurrence analysis, particularly in Cluster 1, which emphasizes swimming performance, stroke techniques, and velocity. In addition, the interdisciplinary connections observed in the categorization analysis, such as biomedical engineering and environmental sciences, are found in the keyword clusters, underscoring the multifaceted nature of swimming research. Specifically, Cluster 2 highlights the nuances of performance and reproducibility among elite swimmers, suggesting opportunities for further investigation of kinematic variables and strategic adaptations in competitive contexts. Cluster 3 delves into the biomechanical intricacies of competitive swimming, providing insights into swimmers' start techniques and variability of success. Additionally, Cluster 4 highlights parameters and strategies relevant to race analysis and performance evaluation, suggesting potential directions for research into elite-level front crawl techniques and underwater strategies. By elucidating these research hotspots, the present study provides a roadmap for future investigations and emphasizes the importance of interdisciplinary collaborations and focused investigations to advance knowledge and innovation in race analysis in swimming.

The main limitation is that only the WoS database was used to identify and screen studies on this topic. This may have neglected some studies on this topic, such as the study by Kennedy and co-workers (105). It should also be noted that some experts on this topic with great international experience did not publish in the aforementioned journals (scientific journals), but rather in another type of publication. However, it should be mentioned that the WoS database is considered to be the oldest, most widely used, and most authoritative database of research publications and citations in the world (28). It covers multidisciplinary topics, high-quality content, and introduces the concept of citation indexing, which allows researchers to track the influence of articles and identify seminal works in a given field. As recommendations for future studies, it can be stated that researchers can gain bibliometric insights into (i) the remaining sports that are held by the Fédération Internationale de Natation (FINA), i.e., open water swimming, diving, water polo, and synchronized swimming, and; (ii) other sports in general or specific topics within each sport.

## 5 Conclusions

This bibliometric review allowed to understand the evolution of the articles published on race analysis in swimming. Since the publication of the first three articles (1984), only since 2012 has there been a sustained increase in publications. The prediction model indicates that the number of articles and citations on this topic will continue to grow until 2034. Sports sciences emerges as the category with the most published articles. Overall, Australia, Spain, and Switzerland are the countries that contribute most to the state of the art in race analysis in swimming until the end of 2023. There are five main clusters of researchers contributing significantly to this topic, with the Swiss, Spanish, and Portuguese groups being the most active in recent years.

## References

[1] Morais JE Barbosa TM Forte P Silva AJ Marinho DA. Young swimmers’ anthropometrics, biomechanics, energetics, and efficiency as underlying performance factors: a systematic narrative review. Front Physiol. (2021) 16(12):691919. 10.3389/fphys.2021.691919 PMC8481572 34603070

[2] Olstad BH Gonjo T Conceição A Šťastný J Seifert L. Arm–leg coordination during the underwater pull-out sequence in the 50, 100 and 200 m breaststroke start. J Sci Med Sport. (2022) 25(1):95–100. 10.1016/j.jsams.2021.08.006 34462220

[3] Sanders R Takagi H Vilas-Boas J. How technique modifications in elite 100 m swimmers might improve front crawl performances to podium levels: swimming ‘chariots of fire’. Sports Biomech. (2023) 22(12):1532–51. 10.1080/14763141.2021.1998590 34766533

[4] van Houwelingen J Willemsen D HJ Kunnen R van Heijst G Grift EJ Breugem WP The effect of finger spreading on drag of the hand in human swimming. J Biomech. (2017) 3(63):67–73. 10.1016/j.jbiomech.2017.08.002 28823502

[5] Cohen RCZ Cleary PW Mason BR Pease DL. Forces during front crawl swimming at different stroke rates. Sports Engineering. (2018) 21(1):63–73. 10.1007/s12283-017-0246-x

[6] Arellano R Brown P Cappaert J Nelson RC. Analysis of 50-, 100-, and 200-m freestyle swimmers at the 1992 Olympic games. J Appl Biomech. (1994) 10(2):189–99. 10.1123/jab.10.2.189

[7] Ruiz-Navarro JJ Cano-Adamuz M Andersen JT Cuenca-Fernández F López-Contreras G Vanrenterghem J Understanding the effects of training on underwater undulatory swimming performance and kinematics. Sports Biomech. (2024) 23(6):772–87. 10.1080/14763141.2021.1891276 33663350

[8] Valkoumas I Gourgoulis V. Sprint resisted swimming training effect on the swimmer’s hand orientation angles. J Biomech. (2024) 164:111991. 10.1016/j.jbiomech.2024.111991 38359622

[9] Arellano R Ruiz-Navarro JJ Barbosa TM López-Contreras G Morales-Ortíz E Gay A Are the 50 m race segments changed from heats to finals at the 2021 European swimming championships? Front Physiol. (2022) 13:797367. 10.3389/fphys.2022.797367 35910554 PMC9326221

[10] Gonjo T Olstad BH. Race analysis in competitive swimming: a narrative review. Int J Environ Res Public Health. (2020) 18(1):69. 10.3390/ijerph18010069 33374118 PMC7795652

[11] Morais JE Marinho DA Arellano R Barbosa TM. Start and turn performances of elite sprinters at the 2016 European championships in swimming. Sports Biomech. (2019) 18(1):100–14. 10.1080/14763141.2018.1435713 29578384

[12] Born DP Lomax I Rüeger E Romann M. Normative data and percentile curves for long-term athlete development in swimming. J Sci Med Sport. (2022) 25(3):266–71. 10.1016/j.jsams.2021.10.002 34764012

[13] Veiga S Roig A. Underwater and surface strategies of 200 m world level swimmers. J Sports Sci. (2016) 34(8):766–71. 10.1080/02640414.2015.1069382 26186108

[14] Peters MD Marnie C Colquhoun H Garritty CM Hempel S Horsley T Scoping reviews: reinforcing and advancing the methodology and application. Syst Rev. (2021) 10(1):263. 10.1186/s13643-021-01821-3 34625095 PMC8499488

[15] Donthu N Kumar S Mukherjee D Pandey N Lim WM. How to conduct a bibliometric analysis: an overview and guidelines. J Bus Res. (2021) 133:285–96. 10.1016/j.jbusres.2021.04.070

[16] Jiang Z Zhao X Wang Z Herbert K. Safety leadership: a bibliometric literature review and future research directions. J Bus Res. (2024) 172:114437. 10.1016/j.jbusres.2023.114437

[17] van Eck NJ Waltman L. Software survey: VOSviewer, a computer program for bibliometric mapping. Scientometrics. (2010) 84(2):523–38. 10.1007/s11192-009-0146-3 20585380 PMC2883932

[18] Chen X Ding R Xu K Wang S Hao T Zhou Y. A bibliometric review of natural language processing empowered mobile computing. Wirel Commun Mob Com. (2018) 18:21. 10.1155/2018/1827074

[19] Yan S Zhang L. Trends and hot topics in linguistics studies from 2011 to 2021: a bibliometric analysis of highly cited papers. Front Psychol. (2023) 13:1052586. 10.3389/fpsyg.2022.1052586 36710766 PMC9875073

[20] Yan S Zhang H Wang J. Trends and hot topics in radiology, nuclear medicine and medical imaging from 2011 to 2021: a bibliometric analysis of highly cited papers. Jpn J Radiol. (2022) 40(8):847–56. 10.1007/s11604-022-01268-z 35344133 PMC8958482

[21] Zhang F Chan AP Li D. Developing smart buildings to reduce indoor risks for safety and health of the elderly: a systematic and bibliometric analysis. Saf Sci. (2023) 168:106310. 10.1016/j.ssci.2023.106310

[22] Cruz AB Kim HD. A bibliometric review of coach leadership studies. Front Psychol. (2023) 8(14):1135243. 10.3389/fpsyg.2023.1135243 PMC9945541 36844359

[23] Hammerschmidt J Calabuig F Kraus S Uhrich S. Tracing the state of sport management research: a bibliometric analysis. Manag Rev Q. (2024) 74:1185–208. 10.1007/s11301-023-00331-x

[24] Zhou T. Bibliometric analysis and visualization of online education in sports. Cogent Soc Sci. (2023) 9(1):2167625. 10.1080/23311886.2023.2167625

[25] Chen XX Ji ZG Wang Y Xu J Wang LY Wang HB. Bibliometric analysis of the effects of mental fatigue on athletic performance from 2001 to 2021. Front Psychol. (2023) 13:1019417. 10.3389/fpsyg.2022.1019417 36698588 PMC9869051

[26] Millet GP Brocherie F Burtscher J. Olympic Sports science—bibliometric analysis of all summer and winter Olympic sports research. Front Sports Act Living. (2021) 20(3):772140. 10.3389/fspor.2021.772140 PMC8564375 34746779

[27] Özkadi T Demir E Yildirim T Çağla Çağlar E Alagöz İ Aydoğdu G. Bibliometric analysis of swimming publications in sports science: a medical perspective. Hitit Med J. (2022) 4(2):39–48. 10.52827/hititmedj.1121920

[28] Birkle C Pendlebury DA Schnell J Adams J. Web of science as a data source for research on scientific and scholarly activity. Quant Sci Stud. (2020) 1(1):363–76. 10.1162/qss_a_00018

[29] Barroso R Crivoi E Foster C Barbosa AC. How do swimmers pace the 400 m freestyle and what affects the pacing pattern? Res Sports Med. (2021) 29(6):598–604. 10.1080/15438627.2020.1860051 33307810

[30] Born DP Bjorklund G Lorentzen J Stoggl T Romann M. Specialize early and select late: performance trajectories of world-class finalists and international- and national-class swimmers. Int J Sports Physiol Perform. (2023) 19(2):164–72. 10.1123/ijspp.2023-0171 38061353

[31] Born DP Kuger J Polach M Romann M. Start and turn performances of elite male swimmers: benchmarks and underlying mechanisms. Sports Biomech. (2024) 4:484–502. 10.1080/14763141.2021.1872693 33663342

[32] Born DP Kuger J Polach M Romann M. Turn fast and win: the importance of acyclic phases in top-elite female swimmers. Sportsbasel. (2021) 9(9):122. 10.3390/sports9090122 PMC8472918 34564327

[33] Born DP Romann M Stoeggl T. Start fast, swim faster, turn fastest: section analyses and normative data for individual medley. J Sports Sci Med. (2022) 21(2):233–44. 10.52082/Jssm.2022.233 35719225 PMC9157519

[34] Born DP Rueger E Beaven CM Romann M. Comparing cross-sectional and longitudinal tracking to establish percentile data and assess performance progression in swimmers. Sci Rep. (2022) 12(1):10292. 10.1038/s41598-022-13837-3 35717501 PMC9206680

[35] Born DP Stacker I Romann M Stoeggl T. Competition age: does it matter for swimmers? BMC Res Notes. (2022) 15(1):82. 10.1186/s13104-022-05969-6 35197115 PMC8867847

[36] Breen D Powell C Anderson R. Pacing during 200-m competitive masters swimming. J Strength Cond Res. (2020) 34(7):1903–10. 10.1519/JSC.0000000000003621 32271289

[37] Burkett B Mellifont R. Sport science and coaching in paralympic swimming. Int J Sports Sci Coach. (2008) 3(1):105–12. 10.1260/174795408784089324

[38] Chow JWC Hay JG Wilson BD Imel C. Turning techniques of elite swimmers. J Sports Sci. (1984) 2(3):241–55. 10.1080/02640418408729720

[39] Cuenca-Fernández F Ruiz-Navarro JJ Polach M Arellano R Born DP. Turn performance variation in European elite short-course swimmers. Int J Environ Res Public Health. (2022) 19(9):5033. 10.3390/ijerph19095033 35564428 PMC9102928

[40] Cuenca-Fernandez F Ruiz-Navarro JJ Polach M Arellano R Born DP. Short-course performance variation across all race sections: how 100 and 200 m elite male swimmers progress between rounds. Front Sports Act Living. (2023) 28(5):1146711. 10.3389/fspor.2023.1146711 PMC10086268 37057072

[41] Daly D Malone L Smith D Vanlandewijck Y Steadward R. The contribution of starting, turning, and finishing to total race performance in male paralympic swimmers. Adapt Phys Activ Q. (2001) 18:316–33. 10.1123/apaq.18.3.316

[42] Demarie S Pycke JR Pizzuti A Billat V. Pacing of human locomotion on land and in water: 1500 m swimming vs. 5000m running. Appl Sci. (2023) 13:6455. 10.3390/app13116455

[43] Escobar DS Hellard P Pyne DB Seifert L. Functional role of movement and performance variability: adaptation of front crawl swimmers to competitive swimming constraints. J Appl Biomech. (2018) 34(1):53–64. 10.1123/jab.2017-0022 28952848

[44] Fischer S Braun C Kibele A. Jason Lezak again and again-linear mixed modelling analysis of change-over times in relay swimming races. J Sports Sci. (2019) 37(14):1609–16. 10.1080/02640414.2019.1578448 30768377

[45] Fortin-Guichard D Ravensbergen HJC Krabben K Allen PM Mann DL. The relationship between visual function and performance in para swimming. Sports Med Open. (2022) 8(1):20. 10.1186/s40798-022-00412-3 35122208 PMC8816996

[46] Fritzdorf SG Hibbs A Kleshnev V. Analysis of speed, stroke rate, and stroke distance for world-class breaststroke swimming. J Sports Sci. (2009) 27(4):373–8. 10.1080/02640410802632623 19235006

[47] Fulton SK Pyne D Hopkins W Burkett B. Variability and progression in competitive performance of paralympic swimmers. J Sports Sci. (2009) 5:535–9. 10.1080/02640410802641418 19219736

[48] Garcia-Hermoso A Saavedra JM Arellano R Navarro F. Relationship between swim start wall contact time and final performance in backstroke events in international swimming championships. Int J Perform Anal Sport. (2017) 17(3):232–43. 10.1080/24748668.2017.1331573

[49] Gatta G Cortesi M Lucertini F Benelli P Sisti D Fantozzi S. Path linearity of elite swimmers in a 400 m front crawl competition. J Sports Sci Med. (2015) 14(1):69–74. 25729292 PMC4306785

[50] Gonjo T Polach M Olstad BH Romann M Born DP. Differences in race characteristics between world-class individual-medley and stroke-specialist swimmers. Int J Environ Res Public Health. (2022) 19(20):13578. 10.3390/ijerph192013578 36294159 PMC9603436

[51] Hogarth L Payton C Van de Vliet P Burkett B. The impact of limb deficiency impairment on para swimming performance. J Sports Sci. (2020) 8:839–47. 10.1080/02640414.2020.1735983 32138613

[52] Lara B Del Coso J. Pacing strategies of 1500 m freestyle swimmers in the world championships according to their final position. Int J Environ Res Public Health. (2021) 18(14):7559. 10.3390/ijerph18147559 34300007 PMC8304102

[53] Lipinska P Allen SV Hopkins WG. Relationships between pacing parameters and performance of elite male 1500-m swimmers. Int J Sports Physiol Perform. (2016) 11(2):159–63. 10.1123/ijspp.2015-0117 26114929

[54] Lipinska P Allen SV Hopkins WG. Modeling parameters that characterize pacing of elite female 800-m freestyle swimmers. Eur J Sport Sci. (2016) 16(3):287–92. 10.1080/17461391.2015.1013996 25703479

[55] Lopez-Belmonte O Gay A Ruiz-Navarro JJ Cuenca-Fernandez F Gonzalez-Ponce A Arellano R. Pacing profiles, variability and progression in 400, 800 and 1500-m freestyle swimming events at the 2021 European championship. Int J Perform Anal Sport. (2022) 22(1):90–101. 10.1080/24748668.2021.2010318

[56] Malone L Sanders R Schiltz J Steadward R. Effects of visual impairment on stroke parameters in paralympic swimmers. Med Sci Sports Exercise. (2001) 33(12):2098–103. 10.1097/00005768-200112000-00019 11740305

[57] Marinho DA Barbosa TM Neiva HP Silva AJ Morais JE. Comparison of the start, turn and finish performance of elite swimmers in 100 m and 200 m races. J Sports Sci Med. (2020) 19(2):397–407. 32390734 PMC7196746

[58] Mauger AR Neuloh J Castle PC. Analysis of pacing strategy selection in elite 400-m freestyle swimming. Med Sci Sports Exerc. (2012) 44(11):2205–12. 10.1249/MSS.0b013e3182604b84 22648344

[59] McCabe C Mosscrop E Hodierne R Tor E. The characteristics of the breaststroke pullout in elite swimming. Front Sports Act Living. (2022) 23(4):963578. 10.3389/fspor.2022.963578 PMC9445308 36081618

[60] McGibbon KE Pyne DB Heidenreich LE Pla R. A novel method to characterize the pacing profile of elite male 1500-m freestyle swimmers. Int J Sports Physiol Perform. (2021) 16(6):818–24. 10.1123/ijspp.2020-0375 33291067

[61] McGibbon KE Shephard ME Osborne MA Thompson KG Pyne DB. Pacing and performance in swimming: differences between individual and relay events. Int J Sports Physiol Perform. (2020) 15(8):1059–66. 10.1123/ijspp.2019-0381 32283539

[62] Miller JA Hay JG Wilson BD. Starting techniques of elite swimmers. J Sports Sci. (1984) 2(3):213–23. 10.1080/02640418408729718

[63] Morais JE Barbosa TM Bragada JA Nevill AM Marinho DA. Race analysis and determination of stroke frequency—stroke length combinations during the 50-m freestyle event. J Sports Sci Med. (2023) 22(1):156–65. 10.52082/jssm.2023.156 36876182 PMC9982526

[64] Morais JE Barbosa TM Forte P Bragada JA de Souza Castro FA Marinho DA. Stability analysis and prediction of pacing in elite 1500 m freestyle male swimmers. Sports Biomech. (2023) 22(11):1496–513. 10.1080/14763141.2020.1810749 33026294

[65] Morais JE Barbosa TM Lopes T Marinho DA. Race level comparison and variability analysis of 100 m freestyle sprinters competing in the 2019 European championships. Int J Perform Anal Sport. (2022) 22(3):303–16. 10.1080/24748668.2022.2054622

[66] Morais JE Barbosa TM Lopes T Simbaña-Escobar D Marinho D A. Race analysis of the men’s 50 m events at the 2021 LEN European championships. Sports Biomech. (2022):1–17. 10.1080/14763141.2022.2125430 36164890

[67] Morais JE Barbosa TM Silva AJ Veiga S Marinho DA. Profiling of elite male junior 50 m freestyle sprinters: understanding the speed-time relationship. Scand J Med Sci Sports. (2022) 32(1):60–8. 10.1111/sms.14058 34551160

[68] Moser C Sousa CV Olher RR Nikolaidis PT Knechtle B. Pacing in world-class age group swimmers in 100 and 200 m freestyle, backstroke, breaststroke, and butterfly. Int J Environ Res Public Health. (2020) 17(11):3875. 10.3390/ijerph17113875 32486151 PMC7313021

[69] Mytton GJ Archer DT Gibson ASC Thompson KG. Reliability and stability of performances in 400-m swimming and 1500-m running. Int J Sports Physiol Perform. (2014) 9(4):674–9. 10.1123/ijspp.2013-0240 24231408

[70] Mytton GJ Archer DT Turner L Skorski S Renfree A Thompson KG Increased variability of lap speeds: differentiating medalists and non-medalists in middle-distance running and swimming events. Int J Sports Physiol Perform. (2015) 10(3):369–73. 10.1123/ijspp.2014-0207 25230099

[71] Neuloh JE Skorski S Mauger L Hecksteden A Meyer T. Analysis of end-spurt behaviour in elite 800-m and 1500-m freestyle swimming. Eur J Sport Sci. (2021) 21(12):1628–36. 10.1080/17461391.2020.1851772 33198590

[72] Nicol E Adani N Lin B Tor E. The temporal analysis of elite breaststroke swimming during competition. Sports Biomech. (2021) 21:1–13. 10.1080/14763141.2021.1975810 34547991

[73] Oliveira JP Marinho DA Barbosa TM Sampaio T Morais JE. Profile of female swimmers competing in the 50 m events at the 2021 LEN European championships. Int J Perform Anal Sport. (2023) 23(2):97–110. 10.1080/24748668.2023.2191393

[74] Pai YC Hay JG Wilson BD. Stroking techniques of elite swimmers. J Sports Sci. (1984) 2(3):225–39. 10.1080/02640418408729719

[75] Perez-Tejero J Veiga S Almena A Navandar A Navarro E. Effect of functional classification on the swimming race segments during the 2012 London paralympic games. Int J Perform Anal Sport. (2017) 17(4):406–17. 10.1080/24748668.2017.1348059

[76] Polach M Thiel D Kreník J Born DP. Swimming turn performance: the distinguishing factor in 1500 m world championship freestyle races? BMC Res Notes. (2021) 14(1):248. 10.1186/s13104-021-05665-x 34193247 PMC8243611

[77] Qiu X de la Fuente B Lorenzo A Veiga S. Comparison of starts and turns between individual and relay swimming races. Int J Environ Res Public Health. (2021) 18(9):4740. 10.3390/ijerph18094740 33946789 PMC8125495

[78] Robertson EY Pyne DB Hopkins WG Anson JM. Analysis of lap times in international swimming competitions. J Sports Sci. (2009) 27(4):387–95. 10.1080/02640410802641400 19214862

[79] Saavedra JM Escalante Y Garcia-Hermoso A Arellano R Navarro F. A 12-year analysis of pacing strategies in 200-and 400-m individual medley in international swimming competitions. J Strength Cond Res. (2012) 26(12):3289–96. 10.1519/JSC.0b013e318248aed5 22222324

[80] Sanchez L Arellano R Cuenca-Fernandez F. Analysis and influence of the underwater phase of breaststroke on short-course 50 and 100 m performance. Int J Perform Anal Sport. (2021) 21(3):307–23. 10.1080/24748668.2021.1885838

[81] Santos CC Fernandes RJ Marinho DA Costa MJ. From entry to finals: progression and variability of swimming performance at the 2022 fina world championships. J Sports Sci Med. (2023) 22(3):417–24. 10.52082/jssm.2023.417 37711703 PMC10499126

[82] Schipman J Sauliere G Le Toquin B Marc A Forstmann N Toussaint JF Involvement in multiple race events among international para and non-disabled swimmers. Front Sports Act Living. (2021) 28(2):608777. 10.3389/fspor.2020.608777 PMC7876089 33585812

[83] Simbana-Escobar D Hellard P Seifert L. Modelling stroking parameters in competitive sprint swimming: understanding inter- and intra-lap variability to assess pacing management. Hum Mov Sci. (2018) 61:219–30. 10.1016/j.humov.2018.08.002 30195170

[84] Skorski S Faude O Rausch K Meyer T. Reproducibility of pacing profiles in competitive swimmers. Int J Sports Med. (2013) 34(2):152–7. 10.1055/s-0032-1316357 22972249

[85] Skucas K Cizauskas G Lagunaviciene N Pokvytyte V. Analysis of 50 m backstroke class S4 disabled swimmers race parameters. Mechanika. (2016) 22(5):444–8. 10.5755/j01.mech.22.5.12743

[86] Taylor JB Santi G Mellalieu SD. Freestyle race pacing strategies (400 m) of elite able-bodied swimmers and swimmers with disability at major international championships. J Sports Sci. (2016) 34(20):1913–20. 10.1080/02640414.2016.1142108 26854943

[87] Thompson KG Haljand R MacLaren DP. An analysis of selected kinematic variables in national and elite male and female 100-m and 200-m breaststroke swimmers. J Sports Sci. (2000) 18(6):421–31. 10.1080/02640410050074359 10902677

[88] Tourny-Chollet C Chollet D Hogie S Papparodopoulos C. Kinematic analysis of butterfly turns of international and national swimmers. J Sports Sci. (2002) 20(5):383–90. 10.1080/026404102317366636 12043827

[89] Veiga S Cala A Frutos PG Navarro E. Comparison of starts and turns of national and regional level swimmers by individualized-distance measurements. Sports Biomech. (2014) 13(3):285–95. 10.1080/14763141.2014.910265 25325772

[90] Veiga S Cala A Frutos PG Navarro E. Kinematical comparison of the 200 m backstroke turns between national and regional level swimmers. J Sports Sci Med. (2013) 12(4):730–7. 24421733 PMC3873664

[91] Veiga S Cala A Mallo J Navarro E. A new procedure for race analysis in swimming based on individual distance measurements. J Sports Sci. (2013) 31(2):159–65. 10.1080/02640414.2012.723130 22989356

[92] Veiga S Mallo J Navandar A Navarro E. Effects of different swimming race constraints on turning movements. Hum Mov Sci. (2014) 36:217–26. 10.1016/j.humov.2014.04.002 24875044

[93] Veiga S Roig A. Effect of the starting and turning performances on the subsequent swimming parameters of elite swimmers. Sports Biomech. (2017) 16(1):34–44. 10.1080/14763141.2016.1179782 27241626

[94] Veiga S Roig A Gomez-Ruano MA. Do faster swimmers spend longer underwater than slower swimmers at world championships? Eur J Sport Sci. (2016) 16(8):919–26. 10.1080/17461391.2016.1153727 26930126

[95] Wadrzyk L Staszkiewicz R Strzala M. Comparison of race performance characteristics for the 50 m and 100 m freestyle among regional-level male swimmers. Appl Sci Basel. (2022) 12:12577. 10.3390/app122412577

[96] Wolfrum M Knechtle B Rüst CA Rosemann T Lepers R. The effects of course length on freestyle swimming speed in elite female and male swimmers—a comparison of swimmers at national and international level. SpringerPlus. (2013) 1(2):643. 10.1186/2193-1801-2-643 PMC3862862 24349949

[97] Wu PPY Babaei T O’Shea M Mengersen K Drovandi C McGibbon KE Predicting performance in 4×200-m freestyle swimming relay events. PLoS One. (2021) 16(7):e0254538. 10.1371/journal.pone.0254538 34265006 PMC8282077

[98] Sheng Y Huiting Z Qiang Z Chenhui L. A corpus-based bibliometric study of highly cited papers in sport sciences. SAGE Open. (2024) 14(1). 10.1177/21582440231225856

[99] O’Donoghue P. *An Introduction to Performance Analysis of Sport.* 1st ed. London: Routledge (2014). 10.4324/9781315816340

[100] Barbosa TM Barbosa AC Simbaña Escobar D Mullen GJ Cossor JM Hodierne R The role of the biomechanics analyst in swimming training and competition analysis. Sports Biomech. (2021) 22(12):1734–51. 10.1080/14763141.2021.1960417 34402417

[101] McGibbon KE Pyne DB Shephard ME Thompson KG. Pacing in swimming: a systematic review. Sports Med. (2018) 48(7):1621–33. 10.1007/s40279-018-0901-9 29560605

[102] Morais JE Barbosa TM Oliveira JP Sampaio T Silva AJ Marinho DA. Normative data of the start in the 50 m events at the 2021 LEN European championships and understanding its relationship with the final race. Eur J Hum Mov. (2022) 49:71–84. 10.21134/eurjhm.2022.49.9

[103] Morais JE Barbosa TM Neiva HP Marinho DA. Stability of pace and turn parameters of elite long-distance swimmers. Hum Mov Sci. (2019) 63:108–19. 10.1016/j.humov.2018.11.013 30508689

[104] Zhang X Estoque RC Xie H Murayama Y Ranagalage M. Bibliometric analysis of highly cited articles on ecosystem services. PloS One. (2019) 14(2):e0210707. 10.1371/journal.pone.0210707 30742632 PMC6370190

[105] Kennedy P Brown P Chengalur SN Nelson RC. Analysis of male and female Olympic swimmers in the 100-meter events. J Appl Biomech. (1992) 17(2):104–9. 10.1123/ijsb.6.2.187 1324101

